# Systemic inflammatory response syndrome following laparoscopic repair of diaphragmatic injury

**DOI:** 10.4103/0972-9941.72604

**Published:** 2010

**Authors:** Philip Umman

**Affiliations:** Department of Surgery, T D Medical College, Alappuzha, Kerala, India

Dear Sir,

A policy of routine exploratory laparotomy for all patients with penetrating injury to the anterior abdominal wall will result in a significant number of negative laparotomies.[1,2] The use of laparoscopy in the setting of trauma and for repair of acute diaphragmatic hernia has been described, though no guidelines are available, as already mentioned in the article.[3] Though bowel exploration with laparoscopy is not as thorough as with laparotomy, secondary features like significant blood in the peritoneal cavity and presence of bilious fluid can be taken as indicators of bowel injury. A high index of suspicion will enable one to convert to open procedure. This would also be based on the surgeon’s experience. However, hemodynamic instability and peritonitis are strong indicators for laparotomy.[4] There are also case reports of diaphragmatic tears being missed on the initial operation.[5] Laparoscopy may also allow better visualization of diaphragmatic injuries.[6]
